# Measured and modeled personal and environmental NO_2_ exposure

**DOI:** 10.1186/1478-7954-10-10

**Published:** 2012-06-09

**Authors:** Emilie Stroh, Ralf Rittner, Anna Oudin, Jonas Ardö, Kristina Jakobsson, Jonas Björk, Håkan Tinnerberg

**Affiliations:** 1 Department of Laboratory Medicine, Division of Occupational and Environmental Medicine, Lund University, Lund, Sweden; 2 Department of Public Health and Clinical Medicine, Occupational and Environmental Medicine, Umeå University, Umeå, Sweden; 3 Department of Earth & Ecosystem Sciences, Division of Physical Geography and Ecosystem Analysis, Lund University, Lund, Sweden; 4 Department of Occupational and Environmental Medicine, University and Regional Laboratories Region Skåne, SE 221 85, Lund, Sweden

## Abstract

**Background:**

Measured or modeled levels of outdoor air pollution are being used as proxies for individual exposure in a growing number of epidemiological studies. We studied the accuracy of such approaches, in comparison with measured individual levels, and also combined modeled levels for each subject’s workplace with the levels at their residence to investigate the influence of living and working in different places on individual exposure levels.

**Methods:**

A GIS-based dispersion model and an emissions database were used to model concentrations of NO_2_ at the subject’s residence. Modeled levels were then compared with measured levels of NO_2_. Personal exposure was also modeled based on levels of NO_2_ at the subject’s residence in combination with levels of NO_2_ at their workplace during working hours.

**Results:**

There was a good agreement between measured façade levels and modeled residential NO_2_ levels (r_s_ = 0.8, p > 0.001); however, the agreement between measured and modeled outdoor levels and measured personal exposure was poor with overestimations at low levels and underestimation at high levels (r_s_ = 0.5, p > 0.001 and r_s_ = 0.4, p > 0.001) even when compensating for workplace location (r_s_ = 0.4, p > 0.001).

**Conclusion:**

Modeling residential levels of NO_2_ proved to be a useful method of estimating façade concentrations. However, the agreement between outdoor levels (both modeled and measured) and personal exposure was, although significant, rather poor even when compensating for workplace location. These results indicate that personal exposure cannot be fully approximated by outdoor levels and that differences in personal activity patterns or household characteristics should be carefully considered when conducting exposure studies. This is an important finding that may help to correct substantial bias in epidemiological studies.

## Background

It is well known that air pollutants resulting from traffic have a negative impact on health, although the effects and threshold levels are still under consideration [1]. One of the reasons for this uncertainty is the problem of correctly estimating individual exposure to air pollutants for large numbers of people and related problems associated with assessing the health effects in large-scale epidemiological studies. Another reason is that different pollutants can have different effects on health. At present, the World Health Organization (WHO) air quality guidelines only focus on nitrogen oxides (NO_x_ and NO_2_) and particulate matter (PM), together with ozone and sulfur dioxide, as markers of air pollution exposure [1]. Out of these, nitrogen oxide and nitrogen dioxide (NO_2_) are the most suitable markers for traffic-generated combustion of the currently regulated pollutants.

Exposure studies involving individual measuring campaigns are time-consuming and expensive, and it is therefore common to estimate exposure based on data from stationary measuring stations and assume that these recorded levels are comparable to individual exposure [2-6]. To improve the ability to relate health effects to traffic-related air pollution, the rather coarse measure “proximity to (major) roads” has been employed in several studies with good result [4,7-12]. An alternative approach is to use geographical information systems (GIS) and emissions databases to model the concentration and dispersion of air pollutants with high resolution in small areas, such as individual residences [13]. However, since people tend to spend between 60% and 80% of their time at their home and the remaining 20% to 40% elsewhere [4], there is considerable temporal and spatial variability in air pollution levels, especially within urban areas [5]. In addition, people are exposed to different levels depending on the spatial and temporal pattern of their activities. The concentration of air pollutants outside an individual’s residence, which is often used as a proxy for individual exposure, may therefore not accurately reflect the individual’s actual exposure. A study by Nethery et al. (2008) concluded that combining work location exposure with home location exposure improved estimates of personal exposure.

Apart from outside concentrations of air pollution there are also other sources for individual exposure to air pollutants such as NO_2_. According to a publication resulting from the EXPOLIS study, the use of gas appliances were one of the strongest and most consistent NO_2_ exposure determinants (together with outdoor concentrations of NO_2_ and workplace location) [14]. Another well known source of NO_2_ is cigarette smoke [15].

Based on these findings, the aims of the present study were:

1) to investigate how accurately a GIS-based dispersion model and emission database can calculate residential outdoor levels of NO_2_,

2) to investigate how well residential outdoor levels of NO_2_ are correlated to measured personal exposure of NO_2_ during a period of seven days, and

3) to investigate whether modeled exposure combining residential and workplace outdoor levels of NO_2_ better reflects personal exposure.

## Methods

### Study area

**Figure 1:**
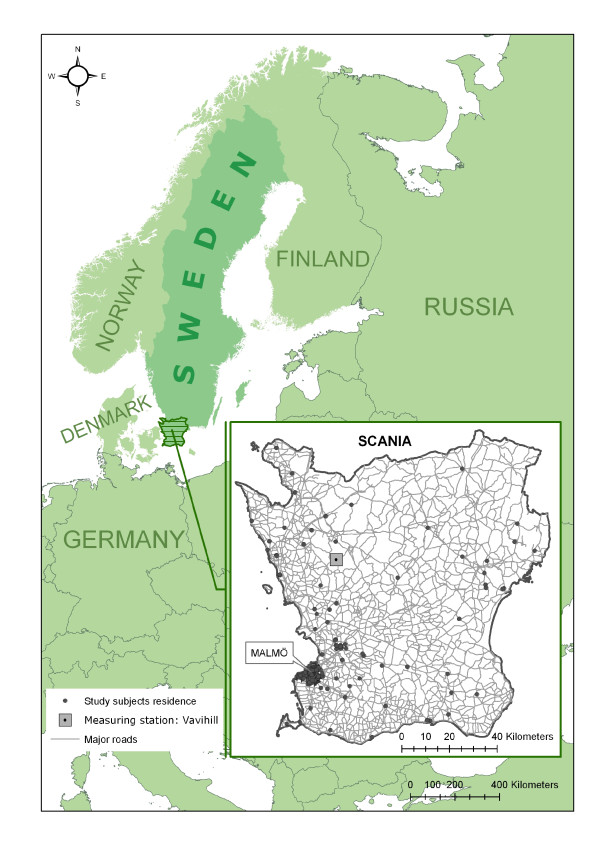
The location of the study area (the county of Scania) in southern Sweden.

The study was carried out in the county Scania in southern Sweden (Figure 1). In Swedish terms, the county is relatively densely populated, with approximately 1.2 million people living in an area of 11,000 km^2^. Sweden’s third largest city (Malmö), with approximately 260,000 inhabitants, is situated in this area. Due to the proximity to the European continent, there is a great deal of cargo transportation to and from this county by road, rail, and water, resulting in high levels of emission. The air pollution levels throughout the county differ considerably due to the geographically varying population density, the proximity to Denmark and its capital Copenhagen, and the vehicle emissions on motorways and other major roads, as well as transportations to and from harbors. Compared with international levels, the levels of NO_2_ in Scania, and Sweden in general, are rather low. Scania has an annual mean level of NO_2_ around 11 to 15 μg/m^3^, while the most populated part of the county (the west coast and the city of Malmö) has an annual mean level of 20 μg/m^3^. The latter value is comparable with the annual mean levels of Sweden’s two biggest cities, Stockholm and Gothenburg, but far from exceeding WHO and European Union guidelines of an annual mean of 40 μg/m^3^. However, these values are urban background values and might be exceeded in hotspots and street canyons. Still, although they are far from exceeding international guidelines of an hourly mean value of 200 μg/m^3^, they still exceed the Swedish environmental quality standard of a daily mean of 60 μg/m^3^[16].

### Study population

The study population was composed of participants in three different measurement campaigns in 2003, 2005 to 2006, and 2008. In the campaigns carried out in 2003 and in 2008, persons aged 20 to 50 and living in the city of Malmö was randomly selected from the national population registry. Letters with information on the study were sent out to 20 to 30 persons at a time, until approximately 40 had agreed to participate (Table 1). There were slightly fewer men than women participating in both studies (45% and 44%, respectively). The mean age was 35 years in 2003 and 34 years in 2008, with a range of 20 to 50 for both years.

**Table 1:** Descriptive statistics of study subjects

		**N**								
			**Sex**							
**Measurement campaign**	**Measurement Period**	**Individuals**	**Men**	**Women**	**Smokers**	**Urban citizens**	**Individuals with gas stoves in their household**	**Personal measurements* (repeated measurement) ***	**Façade measurements* (repeated measurement) ***	**Individuals with information on workplace location**
**2003**	October 6 - November 11	38	17	21	13	38^a^	4	58 *(20)*	-	27
**2005-2006**	September 1, 2005 - June 4, 2006	86	36	50	16	34	5	146 *(60)*	150 *(64)*	56
**2008**	September 22 - December 15	41	18	23	10	41^a^	6	61 *(20)*	-	26

^a^ All individuals lived in the city of Malmö.

The study base for the 2005 to 2006 campaign was a population-based public health survey that was sent to approximately 48,000 persons (aged 18 to 80) living in the county of Scania (return rate of 60%). Individuals from this survey who had given their written approval to be contacted again were divided into two subgroups: individuals with self-reported asthma and controls without asthma. To each asthma case three controls were matched according to gender (in total 3,280 individuals). Another questionnaire was sent investigating more details concerning respiratory health problems and exposure determinants with a response rate of 80% (i.e., 2,616 replies; 580 cases and 2,036 controls). This population was divided into three subgroups, based on the GIS-modeled annual average of NO_2_ at their residence (≤7, 8–12, and >12 μg/m^3^). Fifty cases and 50 controls were randomly chosen from each exposure subgroup, constituting 300 potential participants. The first 100 positive responders were included in the study (in the end 86 individuals took part, out of which 36 were self-reported asthmatics). The gender distribution was similar as in the other studies (slightly fewer men than women took part; 42%) while the mean age was somewhat higher (45 years [range 20 to 67 years]).

### Measured levels of NO_2_

For all of the 165 participants gathered from three different measurement campaigns, the sampling strategy for NO_2_ was similar. The participants carried a diffusion sampler for NO_2_ close to their breathing zone for seven days, providing an integrated measure of NO_2_. One hundred of these participants agreed to take part in an additional week of measurements, providing a total of 265 individual NO_2_ measurements (Table 1). Since gas stoves emit high levels of NO_2_, the data from subjects with a gas stove in their household (N = 15) were removed from the study (in total 24 individual measurements), yielding a total number of 241 measurements.

Different diffusion samplers were utilized to assess personal exposure to NO_2_ in the three measurement campaigns: Willems badge [17] in 2003, a diffusion sampler developed by the Swedish Environmental Research Institute (IVL) [18] in 2005–2006 (hereafter referred to as IVL’s diffusion sampler), and Ogawa [19] in 2008.

The participants were instructed to attach the sampler near the breathing zone, e.g., on their collar, and carry it with them all the time. They were also reminded of the importance of keeping the sampler surface of the monitor unobstructed and in contact with the air to obtain reliable results. They were instructed to remove the sampler when showering, sleeping, or exercising, and put it in a safe, nearby location. In case of rain, the samplers should be temporarily covered.

During the 2005 to 2006 campaign an IVL diffusion sampler (identical to the one used for personal exposure) was attached outside 86 of the participants’ residences during the week of personal monitoring. Sixty-four of the subjects agreed to participate in an additional week of monitoring, providing a total of 150 outdoor measurements at residences (Table 1). The diffusion samplers was placed in an open area about 1.5 meters above the ground, with estimated optimal air turnover and free from unwanted interference, either in the garden of the residence or at the balcony railing if the subject was living in a flat. If possible, the sampler was positioned on the side of the building with less-busy streets. Each sampler was attached to a weather cover to protect it from precipitation.

Since all the subjects did not take part in all the measurements or provide valid workplace information, the number of measurements in the different comparisons varied (Table 1 and 2).

**Table 2:** **Intercepts, slopes, and adjusted r**^**2**^**and p-values from linear regression analysis with 95% confidence intervals (CIs) together with the number of measurements in the analysis (n)**

	**Measured façade levels of NO**_**2**_					
	**Intercept**	**Slope**	**95% CI**	**p**	**r**^**2**^	**n**
**Modeled residential levels of NO**_**2**_	4.7	0.67	0.54-0.80	<0.001	0.42	142
	**Measured personal exposure to NO**_**2**_					
	Intercept	Slope	95% CI	p	r^2^	n
**Measured façade levels of NO**_**2**_						
Univariate	9.1	0.30	0.17-0.44	<0.001	0.12	138
Multivariate^a^	7.1	0.28	0.14-0.41	<0.001	0.19	
**Modeled residential levels of NO**_**2**_						
Univariate	9.7	0.27	0.17-0.36	<0.001	0.11	241
Multivariate^b^	9.2	0.31	0.19-0.43	<0.001	0.12	
**Modeled personal exposure of NO**_**2**_						
Univariate	9.3	0.30	0.17-0.43	<0.001	0.10	165
Multivariate^b^	8.3	0.35	0.17-0.53	<0.001	0.10	

^a^*Adjusted for sex and smoking.*

^b^*Adjusted for sex, smoking, and year of survey.*

### Measured levels of NO_2_

A high-resolution emissions database [20] was used to calculate the hourly levels of NO_x_ (μg/m^3^) during the measurement period for each participant at the location of their residence (N = 165) and at their place of work when data were available (n = 109). The spatial resolution used was 100x100 m. Each modeling session began and ended at noon on the same date as the measuring period began and ended, and both local and regional emission sources were taken into account. The long-range contribution was calculated by modeling local and regional levels of NO_x_ for each measurement period at the location of a regional long-range measurement station (Vavihill, Figure 1) and then subtracting these levels from the recorded levels of NO_x_ (μg/m^3^) measured at the station. The calculated long-range contribution was then added to the previously modeled hourly levels. The hourly NO_x_ levels where then converted into levels of NO_2_ using an equation empirically developed and adjusted for local conditions by the Environmental Department of Malmö City:

(1) $$\begin{array}{l} {\text{NO}}_{2}={\left({\text{NO}}_{\text{x}}\right)}^{(0.74+(28/({\text{NO}}_{\text{x}}}{}^{+153)))} \end{array}$$

(2) $$\begin{array}{l} \left(\text{n}=5548,{\text{ r}}^{2}=0.85\right) \end{array}$$

The hourly levels of NO_2_ were then used to calculate the mean weekly exposure corresponding to each individual measurement using a GIS-program (ESRI® ArcMap 9.2):

a) at the subject’s place of residence, and

b) at the subject’s place of residence and workplace combined.

The level of NO_2_ at the subject’s workplace during workdays (Monday to Friday) and working hours (8 am to 5 pm) were combined with the levels at their residence during the remaining hours of the study period.

### Statistics

PASW Statistics 18 for Windows (Release 18.0.1) was used for all statistical analysis.

Bland-Altman diagrams and linear regression analysis were used to investigate the agreement between:

1) *Measured façade levels of NO*_*2*_ and *Modeled residential levels of NO*_*2*_

2) *Measured personal exposure to NO*_*2*_ and *Measured façade levels of NO*_*2*_

3) *Measured personal exposure to NO*_*2*_ and *Modeled residential levels of NO*_*2*_

4) *Measured personal exposure to NO*_*2*_ and *Modeled personal exposure to NO*_*2*_

Bland-Altman diagrams (or difference plots) are used for visual comparison of the two measurements methods. The differences of the two methods were plotted against the reference or “gold standard” method [21].

Spearman’s rank correlation coefficient was also calculated for these comparisons. Agreement was also assessed separately for nonsmokers.

In a sensitivity analysis, we analyzed data with only one measurement per study subject in order to assess the influence of multiple uses of the same subjects.

In order to investigate the impact of gender, smoking, and year of survey (2003, 2005–2006, and 2008) on agreement, these variables were entered together with the assessed exposure (*Modeled personal exposure, Modeled residential level,* and *Measured façade level*) as independent variables in a multiple linear regression model with *Measured personal exposure of NO*_*2*_ as the dependent variable.

Due to the structure of our data, a paired t-test was calculated for the comparisons of *Measured façade levels* and *Modeled residential levels*. Since our data contained a few outliers we also conducted a nonparametric test (Wilcoxon) for this comparison.

## Results

**Figure 2:**
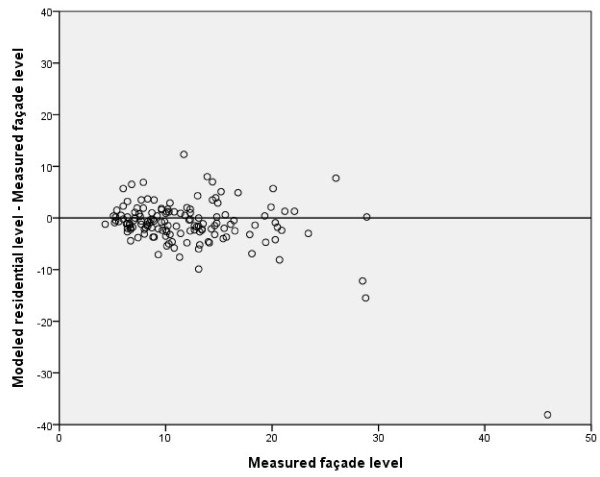
**Bland-Altman plot:*Measured façade level*vs.*Modeled residential level*of NO**_**2**_**(μg/m**^**3**^**); r**_**s**_ **= 0.8, n = 142.**

The association between *Measured façade levels of NO*_*2*_ and *Modeled residential levels of NO*_*2*_ was strong, r_s_ = 0.8, (p < 0.001) (Table 2). The Bland-Altman plot does not indicate any marked systematic difference at any measured level (Figure 2; Table 2). A paired t-test confirmed this finding (mean difference = 1.08 μg/m^3^, 95% CI = 0.28-1.88 μg/m^3^) as well as a nonparametric test (Wilcoxon [p = 0.001]).

**Figure 3:**
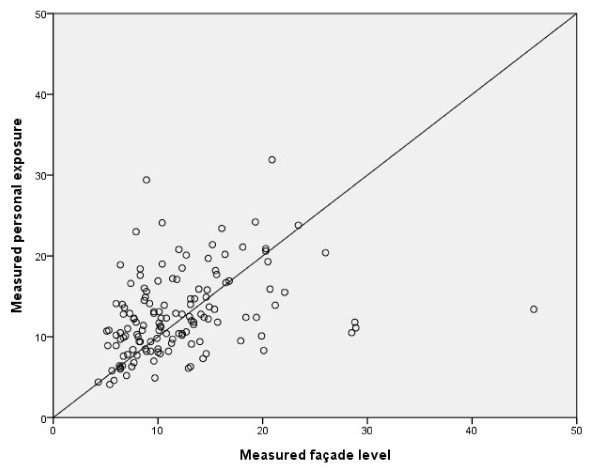
**Correlation between*Measured personal exposure*and*Measured façade level*of NO**_**2**_**(μg/m**^**3**^**); r**_**s**_ **= 0.5, n = 138.**

**Figure 4:**
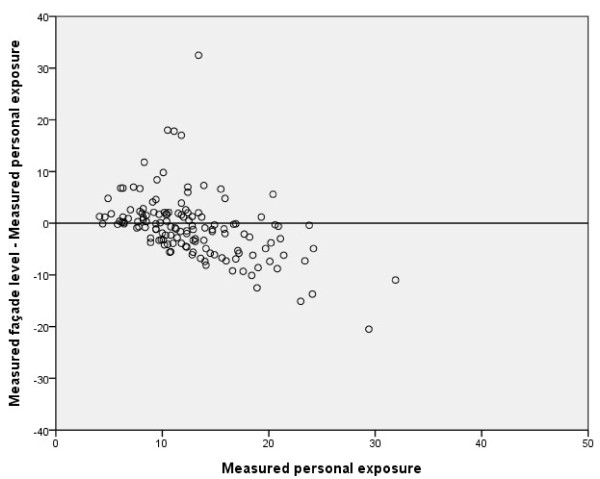
**Bland-Altman plot:*Measured personal exposure*vs.*Measured façade level*of NO**_**2**_**(μg/m**^**3**^**).**

The association between *Measured personal exposure of NO*_*2*_ and *Measured façade levels of NO*_*2*_ was weaker, r_s_ = 0.5 (p < 0.001) (Table 2). Figures 3 and 4 show that façade levels tended to underestimate individually measured levels above 15 μg/m^3^, whereas lower levels were generally overestimated.

**Figure 5:**
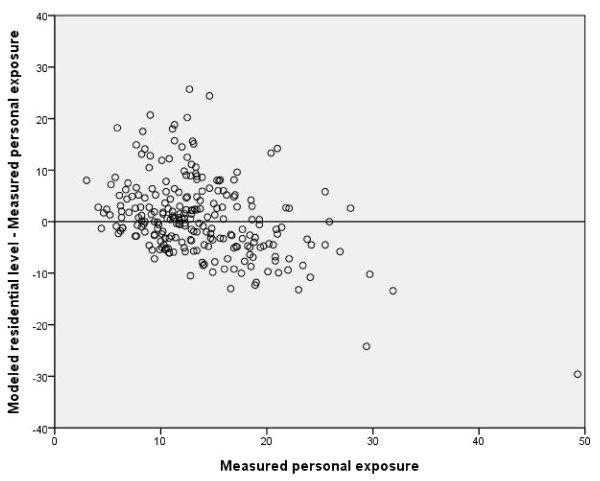
**Bland-Altman plot:*Measured personal exposure*vs.*Modeled residential level*of NO**_**2**_**(μg/m**^**3**^**); r**_**s**_ **= 0.4, n = 241.**

**Figure 6:**
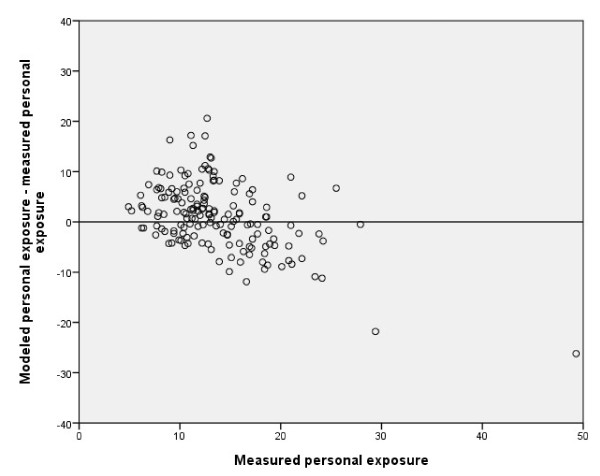
**Bland-Altman plot:*Measured personal exposure*vs.*Modeled personal exposure*of NO**_**2**_**(μg/m**^**3**^**); r**_**s**_ **= 0.4, n = 165.**

The associations between *Measured personal exposure of NO*_*2*_ and the two modeled measures (*Modeled residential levels of NO*_*2*_ and *Modeled personal exposure of NO*_*2*_) were similar: both had a correlation coefficient of r_s_ = 0.4 and a p-value below 0.001 (Figures 5 and 6; Table 2). The modeled levels generally underestimated high individually measured levels, whereas lower levels tended to be overestimated.

The results for nonsmokers were very similar (data not shown) as were the findings of the multivariate analysis (Table 2). Although the variables *Smoking* and *Study 2003* were significant (p < 0.03) in the univariate analysis, neither of these remained significant when included in the multivariate model (data not shown).

The results from the regression analyses in Table 2 remained more or less the same in a sensitivity analysis, excluding subjects with multiple measurements. The intercept increased somewhat for all the regression analyses with approximately 2 to 3 μg/m^3^ (mean 2.6 μg/m^3^, range 1.6 to 3.3 μg/m^3^).

## Discussion

Modeling levels of NO_2_ proved to be a useful method of estimating outdoor concentrations. Our GIS-modeled residential levels of NO_2_ corresponded well with measured façade levels. However, when comparing measured personal exposure with façade levels measured outside the subject’s home, the correlation was significant but low, and even when compensating for workplace location in the model, the agreement between modeled and measured personal exposure was low. Thus, it is possible to model ambient concentrations of air pollutants, provided the data are accurate and of high quality, and the spatial and temporal resolution is sufficiently high. However, these levels do not necessarily reflect personal exposure.

Modeling levels of air pollutants is complex and involves several approximations. The emissions database used in this study gave hourly values of NO_x_, which had to be converted into NO_2_ using an empirically developed equation, which could introduce errors. We only had access to the dates for when the measurements began and had to set up an assumed time for when the corresponding modeling sessions should begin and end (noon).Thus, there is a slight incongruence in time for the measurements and the modeling, which causes some inaccuracies. However, this should not cause any major errors, since most of the modeling sessions lasted for a week, and thus covered the weekly emission patterns near the subjects’ homes. Despite these limitations, the GIS-based model proved to be able to model outdoor levels at the subjects’ residences with sufficient accuracy.

In all our comparisons (Figure 2 to 6) we observed that we had a few outliers with high measured levels of NO_2_. The individuals causing these outliers all lived in major coastal towns with extensive harbor traffic. It is difficult to estimate emissions from harbors, and the emission data in the database may therefore not be completely accurate. Most of the individuals in the lower range of exposure, on the other hand, live in rural “low-level” areas, where partial coverage of the emission sources in the database or a small unrecognized addition in exposure could cause a divergence.

The correlation between measured façade levels of NO_2_ at the subjects’ residences and the measured personal exposure was statistically significant but rather low. This indicates that measurements of outdoor emissions of NO_2_ at the residence are able to predict an individual’s total exposure but that there is a risk for significant misclassification, at least for a mobile population over a period of time as short as a week. Our results are in line with those of previous exposure studies showing that personal exposure often differs significantly from outdoor concentrations [22,23]. In an EXPOLIS study in 2001 [14], investigating the relationship between personal exposure and residential indoor, outdoor, and outdoor workplace location concentrations in Basel, Helsinki, and Prague, it was concluded that gas appliances, outdoor NO_2_ concentrations, and workplace location were the strongest and most consistent NO_2_ determinants. In our study we excluded data from subjects with gas stoves, and tried to compensate for the fact that people live in one area and work in another by using the modeled outdoor levels of NO_2_ at the subject’s workplace during working hours. We had no data regarding the individual’s working hours, so it was assumed that they all worked Monday to Friday, from 8 am until 5 pm. Although this may not apply to all the subjects (e.g., part-time workers, students, and people traveling in their work), it is probably the best approximation. However, the inclusion of workplace location in the model did not improve the correlation between the modeled levels and the measured individual levels of NO_2_.

As can be seen in Figure 6, the modeled personal exposure shows a tendency to overestimate the exposure of subjects with low levels of exposure (<15 μg/m^3^) and underestimate the measured personal exposure for NO_2_ levels above 15 μg/m^3^, similar to the patterns seen in Figures 3 to 5. Since our GIS-based model agreed well with actual measured outdoor levels, we believe that this disagreement is caused mainly by differences in household characteristics or individual time–activity patterns. The reason why outdoor levels tend to overestimate less exposed individuals may be that individuals tend to spend up to 80% of their time indoors [22] where the air quality may differ considerably from that of outdoor air. The major indoor sources of NO_2_ are gas stoves (excluded in this study), active smoking (adjusted for in this study), passive smoking, and the use of fireplaces (not possible to adjust for in this study). Our results are in line with a previous study showing that indoor NO_2_ levels and personal exposure are usually below outdoor levels when no indoor sources of NO_2_ are present [14]. Furthermore, most of the measurements were performed during the winter season when differences in indoor and outdoor levels of NO_2_ are greatest [22]. Most of the sampling was conducted during autumn/winter seasons, with less daylight hours, higher precipitation, and lower temperatures. This likely decreases the population exposure to outdoor levels of NO_2_ since most individuals tend to spend less time outdoors. Thus a larger divergence between measured personal exposure and measured/modeled outdoor levels of NO_2_ can be expected, compared to the summer season.

The divergence between both modeled and measured NO_2_ values for individuals in the upper range (Figures 4 to 6) and the intercepts for the different measures (Table 2) showed that the overall tendency was to underestimate personal exposure, which could not be explained by any pattern regarding home or workplace location (data not shown). These results are contradictory to those reported by Kornatit et al. [22], where the indoor concentrations were significantly lower than outdoor levels but strongly correlated to the personal exposure. Our results do not reflect the same pattern, since our outdoor façade values were generally lower than personal exposure. Since the data from subjects with gas stoves in their homes were removed and neither gender, smoking, nor the different years of the surveys had any substantial influence on the multivariate linear regression coefficients, and including exposure at the workplace did not improve our results, there must be other factors explaining our contradictory results. One could be major indoor sources of NO_2_ that we were unable to control for (e.g., fire places or work exposure). Another much more plausible explanation is differences in individual activity/behavioral patterns that might influence individual exposure to NO_2_ sources. One such factor could be commuting or time spent in environments with high levels of vehicle pollutants. Previous studies have shown that the number of hours spent in a car correlates positively with personal NO_2_ exposure [10]. For other air pollutants such as benzene and PM_2.5_, about 10-15% of the total individual exposure has been estimated to originate from travel [24]. These results may not be fully applicable in our study due to the differences in chemical composition and origin of the pollutants. However, Wheeler et al. [6] showed that concentrations and gradients in outdoor NO_2_ levels were representative of both sulfur dioxide and volatile organic compounds such as benzene and toluene. Zuurbier et al. [25] showed that the mode of transport used for commuting (car, bus, or bicycle) had considerable effects on the concentrations of air pollutants (PM_10_, PM_2.5_, and soot) to which an individual was exposed. Exposure to air pollutants was significantly higher for all modes of transport compared with background levels, but due to the higher breathing rate of cyclists, they inhaled a much higher dose than commuters using other forms of transport [20]. Contrary to these results, Kornatit et al. [22] showed that the NO_2_ levels inside passenger cars were much lower than in all other microenvironments investigated (bedroom, living room, kitchen, office) as well as the outdoor environment.

Since 42% (N = 36) of the participants in the measurement campaign in 2005 to 2006 were self-reported asthmatics, there is a possibility that this study could be affected by selection bias, i.e., the asthmatics might be more aware of the air quality and more prone to avoid areas or situations when they are exposed to elevated levels of air pollution. However, a separate analysis of this group did not show any specific pattern or significant differences compared to the total study population (data not shown).

Our understanding of the impact of emissions in various microenvironments and individual time–activity patterns on personal exposure could perhaps be improved by the use of mobile phones as a personal platform for monitoring exposure to NO_2_ and other air pollutants, while simultaneously registering position and time [26]. However, until further studies on the effects of time spent in traffic and other microenvironments on the personal levels of air pollutants have been conducted, exposure assessment based on modeled or measured levels of outdoor air pollutants for short-term health effect studies should be performed with much caution.

## Conclusion

Emissions databases and GIS-based models proved to be valuable for modeling and estimating outdoor air pollution levels of NO_2_. However, personal exposure measurements of NO_2_ differed significantly from façade levels measured outside the individual’s residence. Taking spatial and temporal differences in outdoor NO_2_ concentrations between the individual’s home and workplace into consideration did not alter these results. Therefore, the use of measured or modeled outdoor air concentrations as a proxy for personal short-term exposure to air pollutants should be undertaken with caution. The low agreement between outdoor levels and personal exposure to air pollution due to individual time-activity pattern that could not be accounted for is a potential source of bias in epidemiological studies. To accurately model personal exposure to air pollutants future research should focus on the modeling of individual time–activity patterns, especially the time spent in traffic, as well as the ability to predict indoor levels from modeled outdoor levels.

## Competing interests

We have no competing interests.

## Authors’ contributions

Idea and general design of the study was conceived by KJ, HT, JA and ES. Data modeling and analysis was done by ES and RR. Statistic was performed by ES and AO with guidance from JB. The paper was written by ES with advice and comments from HT, JA, KJ, AO and RR. All authors read and approved the final manuscript.
